# Influence of Incubation Time for Viability Assessment of *Ascaris suum* Eggs—Attempts to Optimise the Parasitological Examination

**DOI:** 10.3390/pathogens14101070

**Published:** 2025-10-21

**Authors:** Radosław Badziak, Jolanta Zdybel, Zbigniew Osiński, Ewa Bilska-Zając, Jacek Karamon, Jacek Sroka, Marta Skubida, Angelina Wójcik-Fatla, Tomasz Cencek

**Affiliations:** 1 Department of Parasitology and Invasive Diseases, Bee Diseases and Aquatic Animal Disease, National Veterinary Research Institute in Puławy, Al. Partyzantow 57, 24-100 Puławy, Poland; radoslaw.badziak@piwet.pulawy.pl (R.B.); ewa.bilska@piwet.pulawy.pl (E.B.-Z.); j.karamon@piwet.pulawy.pl (J.K.); jacek.sroka@piwet.pulawy.pl (J.S.); marta.skubida@piwet.pulawy.pl (M.S.); tcencek@piwet.pulawy.pl (T.C.); 2 Department of Virology and Viral Diseases of Animals, National Veterinary Research Institute in Puławy, Al. Partyzantow 57, 24-100 Puławy, Poland; zbigniew.osinski@piwet.pulawy.pl; 3 Department of Health Biohazards and Parasitology, Institute of Rural Health, Jaczewskiego 2, 20-090 Lublin, Poland; afatla@poczta.onet.pl

**Keywords:** *Ascaris suum* eggs, incubation, parasitological methods, sewage sludge, viability assessment

## Abstract

The aim of this study was to determine the optimal incubation time for accurate assessment of *Ascaris suum* eggs viability, an important step in improving parasitological diagnostics. The experiment used *A. suum* eggs collected from three sources: adult roundworms uteri (U), pig faeces (F) and sewage sludge (S), then incubated at 27 °C and monitored weekly. The number of Petri dishes used for eggs observations for each source was 12 (100 eggs were observed on each Petri dish). Eggs were classified as dead (with clear deformations), viable (with motile larvae) or of uncertain viability (retaining structural integrity but undeveloped). The results showed that eggs from group U had the highest viability (96%) and developed larvae within 3 weeks. In contrast, group F (52% viability) and S (3% viability) showed delayed development, requiring up to 8–12 weeks for a conclusive viability assessment. The differences in the percentage of QE, LE, and DEwere generally statistically significant, except in the case of eggs from sewage sludge in the fourth week of incubation. These results indicate significant differences in egg viability depending on the sample source and emphasise the need for longer incubation times, particularly for environmental samples such as sewage sludge. The study also highlights the limitations of single time point assessments based solely on egg structure, which can lead to misclassification. In conclusion, prolonged incubation improves diagnostic accuracy by allowing a clearer distinction between viable and non-viable eggs, especially in samples with initially uncertain viability.

## 1. Introduction

The regulations in the European Union and in Polish law impose an obligation to assess the sanitary condition of organic fertilisers and arable land fertilised with such substances [1,2,3]. One of the indicators of the hygienic condition of soils and organic fertilisers is the presence of live intestinal parasites eggs of the genera *Ascaris*, *Toxocara* and *Trichuris* [4,5].

Diagnostics of a parasitic infection in humans or animals by the detection parasite developmental stages, e.g., eggs in faeces, is considered the most common and relatively reliable diagnostic method [6]. In routine diagnostics (e.g., coproscopic methods) the viability of helminth eggs is irrelevant, as the presence of unfertilised or dead eggs in faeces also confirms the presence of infection of the organism (human or animal) by a parasite. However, in the parasitological analysis of sewage sludge, soil or organic fertilisers, it is also necessary to determine the viability of the eggs. This is because the purpose of testing these materials is to assess the risk to human and animal health.

Laboratories use various parasitological methods to assess the viability of intestinal parasite eggs, which can generally be classified into three groups:

1. Methods based on the staining of eggs with fluorescent dyes (e.g., LIVE/DEAD BacLight Bacterial Viability KitLIVE (Molecular Probes, Invitrogen, Eugene, OR, USA)) [7,8,9].
2. Methods involving a visual assessment of the integrity of the internal structures of the eggs without an incubation process (e.g., AFNOR XP X33-040: Characterization of sludges-Enumeration and viability of parasite helminth eggs-Triple flotation technique) [10,11,12,13].
3. Methods involving incubation of eggs and periodic observation of changes in their internal structures [14,15,16,17,18,19,20,21].

Each of these methods has advantages and limitations. Methods based on egg staining are highly sensitive and are particularly suitable for assessing the viability of eggs isolated from the uteri of adult female nematodes. However, in the case of eggs from sewage sludge, staining results may be inconclusive. This is due to the possible staining of bacteria that inhabit the surface of the eggs, which can interfere with proper analysis [7,9]. Methods relying solely on visual assessment of the internal structures of eggs are fast. However, they carry a high risk of error. Although they only require a single observation under the microscope, they are prone to misdiagnosis. According to our observations, dead eggs can retain normal structural features for a long period of time, making an accurate assessment of viability difficult. Conversely, methods based on incubation and microscopic evaluation offer greater certainty of classification by observing embryo degradation or development during incubation. However, they are also susceptible to misclassification because the development or degradation of helminths eggs can be very slow (e.g., eggs obtained from sewage sludge). The generally accepted incubation conditions (3 weeks at 27 °C) may not be adequate in such cases.

The aim of this study was therefore to determine the incubation time required for an ac-curate and efficient assessment of the viability of *Ascaris suum* eggs. This parasite was chosen due to the frequent occurrence of its eggs in fertilisers, sewage sludge, wastewater and soil [22,23,24]. It was also chosen because eggs and adult forms are easily obtained for experiments.

## 2. Materials and Methods

### 2.1. Parasite Eggs

The experiment utilised *Ascaris suum* eggs collected from three sources: adult nematodes uteri (U), pig faeces (F) and sewage sludge (S).

### 2.2. The Process of Obtaining Eggs from the Uterus of Mature Female Nematodes (U)

Adult female *A. suum* nematodes were harvested from the intestines of naturally infected pigs at a slaughterhouse. Nematodes were classified as *A. suum* based on their morphological characteristics [25]. The eggs were then isolated from the terminal segment of the uterus of 10 adult females *A. suum* worms. This isolation was carried out according to our own procedure. Using surgical instruments, the entire uterus was carefully extracted from the nematodes. The distal uterine segments, which measured between 1.5 and 2 cm in length, were then passed through a sieve with sieve opening 1 mm to pre-clean the egg suspension. The eggs were then placed in distilled water using a pipette and sonicated for 15 min in an ultrasonic cleaner (Badelin Sonorex, Bandelin electronic GmbH & Co. KG, Berlin, Germany) to break up any conglomerates. The resulting suspension was stirred with a magnetic stirrer (IKA BIG SQUID, IKA Werke GmbH & Co. KG, Staufen, Germany) for 10 min. After adding a 1% formaldehyde (Avantor Performance Materials Poland Inc., Gliwice, Poland) solution, the suspension was stored at approximately 4 °C for no longer than seven days prior to the experiment.

### 2.3. The Process of Isolating Eggs from Pig Faeces (F)

The eggs were isolated from the faeces of pigs infected with *A. suum* and slaughtered at a single abattoir. They were isolated by flotation in a saturated magnesium sulphate (MgSO_4_) (Chempur Ltd., Piekary Śląskie, Poland) solution combined with centrifugation (own method). The faecal sample was thoroughly mixed with the saturated MgSO_4_ solution (specific gravity 1.28) and filtered through a 200 µm sieve. It was then centrifuged (Sigma G-16KS, Sigma Laborzentrifugen GmbH, Osterode am Harz, Germany)for 10 min at 2500× *g*. After centrifugation, the upper portion of the supernatant was carefully poured into a glass beaker and the eggs were transferred using a automatic pipette (Thermo Fisher 0,5-5 µl, Thermo Fisher Scientific, Waltham, MA, USA) to a Petri dishes containing distilled water and 1% formaldehyde (Avantor Performance Materials Poland Inc., Gliwice, Poland) solution. The resulting suspension was stored at approximately 4 °C for no longer than seven days prior to the experiment. Eggs were classified as *A. suum* based on their morphological characteristics [25]. The faecal samples were free from contamination with eggs of other parasites.

### 2.4. The Process of Isolating Eggs from Dewatered Sewage Sludge Obtained from a Wastewater Treatment Plant (S)

The isolation of eggs from dewatered sewage sludge obtained immediately after dehydration from a municipal wastewater treatment plant in Poland was conducted using own method previously described [19] and standardised as PN-Z-19005:2018-10 [19]. The sample was mechanically mixed with a 0.0025% Tween 20 (Chempur Ltd., Piekary Śląskie, Poland) solution for four hours and then filtered through a 200 µm sieve. The filtrate then underwent centrifugationfor 10 min at 2500× *g*, after which the supernatant was removed. The resulting precipitate was then mixed with saturated sodium nitrate (NaNO_3_ (Chempur Ltd., Piekarty Śląskie, Poland)) (specific gravity 1.35) and subjected to another round of centrifugation for 10 min at 2500× *g*, after which it was sedimented in distilled water for 1.5 h. After sedimentation, the precipitate containing the isolated eggs (in distilled water and a 1% formaldehyde solution) was stored at approximately 4 °C for no longer than seven days prior to the experiment [21]. Eggs were classified into the *A. suum* category based on their morphological characteristics [25]. Although eggs belonging to other intestinal parasite species (e.g., *Toxocara* spp. and *Trichuris* spp.) were observed in sewage sludge samples, only *A. suum* eggs were isolated using an automatic pipette.

### 2.5. Experiment: Estimation of Egg Viability

Isolated eggs were transferred separately into 95 mm diameter Petri dishes containing 1% formaldehydesolution using an automatic pipette (200 eggs per dish). The eggs were isolated using an automatic pipette. Each egg was transferred individually to a Petri dish. Each plate was numbered. Three types of cultures were prepared:

- 12 plates with eggs isolated from the uterus of adult female *Ascaris suum* (group U),
- 12 plates with eggs isolated from pig faeces (group F),
- 12 plates with eggs isolated from sewage sludge (group S).

After distribution of the eggs into Petri dishes, an initial viability observation was made using a stereoscopic microscope (Olympus SZX2-ILLTS, OLYMPUS, Tokyo, Japan) (40–100× magnification). The Petri dishes were then placed in a thermostat (Memmert IPP 300, Memmert GmbH & Co. KG, Schwabach, Germany) and incubated at a temperature range of 26–28 °C. The level of the solution in the dish was marked with a pen to provide a reference point. 1% formaldehydesolution was added to maintain the desired level in case of evaporation. Based on our observations [21] and the current standards [19,20] and literature [14,26], a temperature of 26–28 °C was selected for this study. The incubator’s temperature was monitored and recorded daily, and the suspensions in the dishes were mixed by hand to allow oxygenation to proceed. The plates were observed weekly for 12 weeks. The necessary incubation time was defined as the time required for all eggs of uncertain viability to disappear from the pool of incubated eggs (i.e., to develop or degrade).

At each observation, 100 eggs (being a representative sample of the 200 placed on each plate) were counted, observed and classified into one of three categories based on morphological changes to the eggshell:

- eggs with clear deformations (such as granular appearance, deformed cytoplasm, damaged shell or empty shell) were classified as dead eggs (DE),
- eggs in which motile larvae had developed were classified as live eggs (LE),
- eggs that retained correct structural features but did not show signs of embryo development (such as cleavage or larval development) were classified as eggs of questionable viability (QE).

Eggs containing a non-motile larva that was observed to degrade during incubation were also considered dead. In the following weeks of observation, the percentages of each egg class were calculated. These percentages included eggs from various sources (U, F, S) categorised into different classes (QE, LE, DE) at each survey date for comparison.

The time required to correctly assess egg viability was the time when all incubated QE eggs could be classified as LE or DE class.

### 2.6. Statistical Analysis

Statistical analysis of the results was performed using STATISTICA 13 software (StatSoft, Kraków, Poland). Multiple regression models were applied to estimate interactions between the proportions of QE, LE, and DE over time.

The normality of variable distributions in the uterus (U), faeces (F), and sludge (S) groups was assessed using the Shapiro–Wilk test. Since the assumption of normality was not met, a non-parametric rank-based MultiANOVA test was used for further comparisons.

To identify statistically significant differences between the groups, Tukey’s Honest Significant Difference (HSD) post hoc test was applied. A significance level of α = 0.05 was adopted for all statistical procedures.

Additionally, Z-scores and Student’s *t*-tests were calculated to evaluate whether the week-to-week changes in the proportion of each egg category (QE, LE, DE) were statistically significant in relation to the group means. These tests allowed us to determine whether the observed dynamics were the result of real effects rather than random variation.

## 3. Results

During the experiment (12 weeks), all types of *A. suum* eggs (QE—eggs of unconfirmed viability, LE—eggs with live larvae and DE—degraded eggs) were observed in all samples taken from the roundworms uterus, pig faeces and sewage sludge. However, the dynamics of their appearance varied significantly between the groups. Table 1, Table 2 and Table 3 present the results (with statistical analysis).

Further statistical analyses are presented in Tables S1–S10, which can be found in the Supplementary Materials.

**Figure 1:**
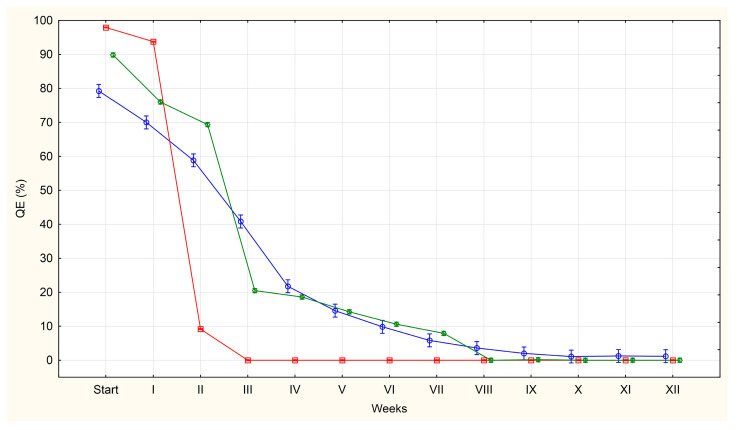
The average percentages of *A. suum* eggs of questionable viability (QE) isolated from the uteri of adult female roundworms (U)—red line, obtained from pig faeces (F)—green line and from sewage sludge (S)—blue line.

**Figure 2:**
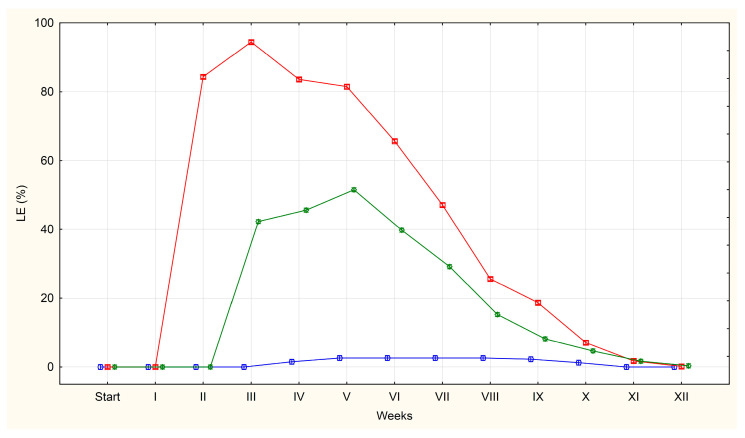
The average percentages of *A. suum* eggs containing live larvae (LE) isolated from the uteri of adult female roundworms (U)—red line, obtained from pig faeces (F)—green line and from sewage sludge (S)—blue line.

**Figure 3:**
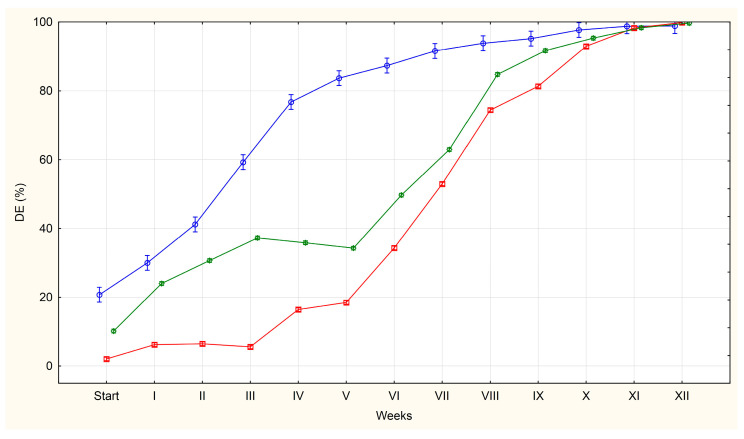
The average percentages of *A. suum* eggs classified as dead (DE) isolated from the uteri of adult female roundworms (U)—red line, obtained from pig faeces (F)—green line and from sewage sludge (S)—blue line.

The average percentages of QE, LE and DE in each group (U, F, S) are shown in Figure 1, Figure 2 and Figure 3.

At the initial observation point (day 0), only QE and DE were present. Among eggs from the uterus, 98% were classified as QE on average, compared to 90% for eggs from faeces and 79% for eggs from sludge. Conversely, DE were most prevalent in the S group (21%) and least prevalent in the U group (2%). No LE were observed in any group at this stage.

LE (those with the larva were developed) for the first time were detected in the second week of incubation on plates containing eggs isolated from *A. suum* uteri (84% of the total pool of observed eggs). The high proportion of eggs indicates a rapid increase in larval development during the second week. A similar trend was observed one week later in the faecal group, where the percentage of LE increased from 0% to 42%. On plates containing eggs isolated from sewage sludge, the first appearance of LE occurred in the fourth week, with only isolated cases being observed (2%).

In group U, the percentage of eggs containing live larvae remained high for 3–4 weeks (84%, 94%, 84% and 82%), before declining steadily as the larvae died inside the eggs. By week 11, the average had fallen to 2%, and LE were no longer observed by week 12.

Group F followed a similar pattern with LE remaining relatively stable over 3–4 weeks (42%, 46%, 52%, 40%) before declining systematically. By week 11, the average was 2%, dropping to 0% by week 12.

For eggs obtained from sewage sludge, the average percentage of LE remained low following their appearance in week 4, peaking at just 3%. This level was maintained for five weeks, with the eggs disappearing completely by week 11.

Eggs classified as dead (DE) included those with abnormal structure showing signs of degeneration, as well as those in which larval death had occurred. Relatively few such eggs were observed at the first observation (day 0), particularly in groups U (2%) and F (10%), while till 21% were found in group S.

At subsequent observation dates, the percentage of eggs classified as DE increased gradually. The slowest increase was observed in group U, while the fastest increase was observed in group S.

In group S, the percentage of such eggs reached 77% after four weeks of incubation. In groups U and F, it was 16% and 36%, respectively, at the same time point. More than 90% of the DE in groups U, F and S were observed from weeks 10, 9 and 7, respectively. By week 12, almost all the eggs in all the groups were classified as DE.

However, for the purposes of this experiment, the most important class of eggs for diagnostics was defined as QE. Eggs in this group present a particular diagnostic challenge as it is not possible to definitively assess their viability by microscopic examination. Therefore, the results of the statistical analysis will only be described for this class of eggs.

An increase in the number of LE and DE class eggs is accompanied by a systematic decrease in the number of QE class eggs. The most pronounced decrease in QE eggs was observed in group U, where none of these eggs were present by week 3. Tukey’s post hoc test showed that the *p*-values for the comparisons between week III and subsequent weeks (IV–XII) were all greater than 0.05 (*p* = 1.00000), indicating no significant change. In group F, the decrease was slower and QE eggs were not found until week 8 of incubation. All *p*-values for comparisons between week VIII and weeks IX–XII (Tukey’s HSD test) also exceeded 0.05 (*p* = 1.00000), indicating that there was no statistical significance in this phase of incubation. In group S, the decrease in the percentage of QE eggs was even slower, with only a few of these eggs being found from week 10 onwards. However, most of the *p*-values calculated for comparisons between week VII and subsequent weeks (weeks VIII–XII) were greater than 0.05, often reaching a value of approximately 1.00000. This indicates that there were no significant statistical differences in these comparisons. Additionally, the most significant decrease in QE eggs occurred during the first four weeks of incubation across all groups (U, F and S).

A sharp and statistically significant decline in the percentage of QE eggs observed in both the U and F groups (*p* = 0), was confirmed by strongly negative Z-scores and high absolute t-values. Concurrently, a large increase in the percentage of LE (which decreases in the later weeks of incubation) and DE was recorded, with both of these trends also being statistically significant (*p* = 0). This was accompanied by strong positive shifts in statistical indicators.

By contrast, in the sewage sludge group, the decline in the number of QE eggs was gradual but statistically significant in almost all weeks, except week 4 where the analysis showed *p* = 0.25. The lack of significance in week 4 indicates a temporary stabilisation of the decline in the percentage of these eggs. Conversely, statistical significance for LE was only found in weeks 5–8 (by which time their percentage had reached 3%). The percentage of DE increased statistically significantly throughout the incubation period, except in week 4 (*p* = 0.39), when growth slowed temporarily, as evidenced also by increasing Z-score and t-value.

## 4. Discussion

For the experiment the eggs of the species *A. suum* were selected. This decision was based on the common prevalence of these eggs in the environment (such as soil) [27,28], organic fertilisers [29], sewage sludge and digestates used for fertilisation purposes [30,31]. In addition, the host of this parasite are pigs, which are commonly raised for meat production, making it relatively easy to obtain adult forms of nematodes [32,33,34]. In contrast, eggs and adult forms of other parasites described as indicators of sanitary status of fertilisers, sewage sludge or soil (such as *Trichuris* spp. and *Toxocara* spp.) are more challenging to obtain in large quantities. *Toxocara*, for example, are typically found in the intestines of carnivorous animals such as dogs and can only be obtained incidentally during necropsy of deceased animals. In contrast, the prevalence of *Trichuris* spp. infection in pigs is much lower than in the case of *Ascaris* spp. [35]. It is even more difficult to obtain eggs of these parasites from sewage sludge or organic fertilisers as they are rarely found in abundance in these matrices, and obtaining them in sufficient quantities for experiments such as ours is practically impossible. Zdybel et al. (2019) showed in their study, that eggs of nematodes belonging to the genera *Toxocara* and *Trichuris* are several times less abundant in sewage sludge than those of *Ascaris* spp. [30]. Similar results have also been reported by Horak P. in his study carried out in 1992 [36].

Based on the appearance of the different egg categories in our experiment, we identified three distinct periods: an initial period (day 0), during which the ratio of QE, LE and DE illustrates their occurrence in a given matrix; a period during which larvae appeared and developed in the living eggs; and a period during which the larvae in the eggs died. Throughout the experiment, the percentage of QE eggs falls to values close to zero.

On the day 0 QE and DE were observed in all groups. However, in group U only 2% of the eggs were classified as DE, whereas in group F this percentage was 10% and in group S even 21%. These differences in the percentages of QE and DE between all groups can be attributed to the influence of environmental conditions. Eggs isolated from the uteri of adult female roundworms were not exposed to adverse negative external factors. The capacity of dead eggs observed in this group may be due to lack of fertilisation or natural death within the reproductive tract, possibly due to metabolic or genetic disorders [37,38].

The percentage of eggs classified as QE in group F was lower (90%). This difference may be explained by the fact that, after leaving the uteri of *A. suum* females, the eggs come into contact with the digestive system of the host where the presence of microorganisms (such as bacteria or protozoa), digestive enzymes or bile acid salts may have a detrimental effect on the eggs, particularly those with microdamage to the shell layers. However, it is important to note that this explanation is based on our assumption, as there is no available literature data to support this hypothesis. On the contrary there are literature data suggesting that the passage of eggs through the digestive system confers properties of resistance to external environmental conditions and influences the normal development of the larvae [39] but this may only apply to eggs with an undamaged shell structure. It has also been reported that slurry itself has protective properties for *Ascaris* spp. eggs [40].

Among eggs obtained from sewage sludge, we observed even less QE, with the percentage being 79%. In this matrix, after leaving the uteri and surviving the pressures of the host digestive environment, parasite eggs are additionally exposed to sludge conditioning and hygienization processes, which may further reduce the proportion of eggs with a normal structure [5,41]. The literature reports also suggest that higher pH or increased ammonia levels (common in sewage sludge or organic waste treatment processes) can induce eggs destruction [42,43].

The next stage in the incubation process that we identified was the emergence of eggs containing developing live larvae. The development of the larval form was observed in eggs obtained from all sources (U, F, S). The appearance of such eggs was accompanied by a decrease in QE egg percentage and a gradual increase in DE egg percentage (degradation of QE eggs). However, in each group there was a difference in the dynamics of the decrease or increase in the percentage of each egg class over the period.

The shortest time required for larvae development in eggs was observed in those isolated from the uteri of roundworms. There are data in the literature showing that eggs extracted from roundworm uteri may not exhibit normal embryogenesis and may not achieve full infectivity. It is suggested that these eggs may not be fully developed in terms of the composition of the outer egg shell and possibly other features acquired during normal egg excretion and subsequent passage through the host digestive system [44]. Our results did not confirm this data, as almost all eggs isolated from the uteri showed embryonic development. It is important to note that we only obtained eggs from the distal segment of the uteri of adult female roundworms, thereby reduced the problems associated with incomplete egg development. Similar observations to ours were reported by Geenen et al. in 1999 [45]. In their study, the larval form appeared in the eggs after 17–22 days of incubation, although at lower temperatures (18–22 °C). They also presented data indicating that after 4 weeks of incubation, the majority of eggs contained mobile larvae, with less than 9% of eggs remaining unfertilised. Similarly, Maya et al. (2019) [46] conducted a study in which incubation was performed at temperatures of 28 °C and 34 °C. They reported that *A. suum* eggs developed into the larvae after 16–20 days, regardless of the incubation temperature. Furthermore, Jeska et al. (1986) [38] observed that 90% of the eggs contained larvae after 30–35 days. These results are consistent with our observations in the U group.

In eggs from pig faeces (group F), the first larvae were observed somewhat later, appearing between the second and third week of incubation. Similar experiments were carried out by Oksanen et al. (1990) [39]. These authors found, in agreement with our observations, that in *A. suum* eggs isolated from the uteri of adult roundworms and obtained from the faeces of infected pigs developed larvae in the third week of incubation. However, they observed that the percentage of eggs that developed into larvae-containing forms was similar in both groups (88–97%), whereas in the present study, only 52% of eggs isolated from faeces developed into larvae. Several factors could influence this difference, such as the housing conditions, the health status of the pigs from which the faeces were obtained, and their diet. Literature data suggest that the diet provided to the pigs can influence the pH level of the slurry. High pH levels can increase the inactivation of parasite eggs [47].

Eggs isolated from roundworm uteri and eggs obtained from pig faeces remain differently to eggs isolated from sewage sludge. In the last group, the larval forms appeared later, typically between the 3rd and 4th week of incubation.

An analysis of the available literature show that no studies directly comparable to ours have been carried out, making it difficult to refer to the results of other researchers. However, it is highly probable that the delayed development of larval forms and their significantly lower percentage in sewage sludge samples can be attributed to the technological processes employed in wastewater treatment plants. Our own observations [5] support this assumption, indicating that such processes, especially anaerobic processes such as fermentation, as well as hygienization methods like liming or temperature treatment [48,49], can lead to embryo mortality in the majority of parasite eggs.

Simultaneously with the appearance of LE and increase in the number of eggs classified as DE a natural reduction in the number of QE eggs was observed. Among the eggs obtained from the uteri, the number of eggs classified as QE decreased to 9% after the second week of incubation (84% of these eggs developed into LE, while 5% of the eggs were degraded). In eggs obtained from faeces, the percentage of QE eggs decreased to 21% after week 3 (42% of eggs developed into LE and 27% of eggs were degraded). Conversely, in eggs isolated from sludge, this percentage was 22% after four weeks of incubation (in 2% of the eggs developed larvae and as many as 55% degraded).

The third stage highlighted in our experiment was the period when the larvae inside the eggs die and at the same time the percentage of QE and LE decreases to values close to zero. A slow decrease in the percentage of eggs containing live larvae from the uteri was observed from the fourth week of incubation. This process continued over a nine-week period, with the percentage of eggs containing living larvae reaching 0% by week 12 of the experiment. In the case of eggs obtained from faeces, a decrease in the percentage of eggs containing live larvae was observed after week 6 and continued for seven weeks reaching 0% by week 12 of the experiment. In turn, the percentage of eggs containing live larvae in the eggs obtained from sewage sludge remained at 2–3% for another eight weeks before reaching 0% by week 11 of incubation.

It is surprising that the larvae in incubated eggs die off relatively quickly. The short survival of larvae observed in our study, up to 12 weeks, is difficult to compare with the survival of eggs in the natural environment. For example, the survival of *Ascaris* spp. eggs in soil has been reported for up to 10 years [50,51]. However, it is important to note that in our experiment the eggs were still incubated at 27 °C in distilled water after development into larvae. These conditions favour rapid development but not long survival and are very different from those found in the natural environment, such as slurry or soil.

It is interesting to note that in group S, despite the low percentage of eggs in which larval development occurred, eggs with correct structure (as assessed microscopically) persisted for a relatively long time. This phenomenon could possibly be attributed to the physico-chemical and biological processes observed during sludge treatment. These factors could have led to embryo death within the eggs [5]. At the same time, however, they may have preserved the eggs in some way, perhaps by impregnation and inhibiting the degradation of structures inside the eggs shells, thus preventing visual detection of degeneration.

At the end of the discussion, based on the results obtained, we can draw the following practical conclusion from the statistical analysis: the lack of statistical significance in the weekly comparisons can be considered the point at which incubation ends, i.e., the time required to reliably assess egg viability. For group U it this will be the third week, for group F, the eight week; and for group S the seventh week of incubation. Although QE eggs were still present in group S up to week 11th, the lack of larval development in these eggs and the insignificant differences between weeks suggest that seven weeks can be considered the optimal incubation period for assessing viability.

## 5. Conclusions

As shown in our study (both eggs observation and statistical analysis of the results), an incubation period of 3 weeks is sufficient to assess the viability of eggs isolated from roundworm uteri, which are unaffected by external environmental factors. However, for eggs obtained from faeces and sewage sludge, a much longer incubation period (minimum 7–8 weeks) is required for an accurate viability assessment. Therefore, there is a need to re-evaluate the parasitological methods used, especially for the analysis of organic fertilisers produced from sewage sludge, as well as sewage sludge when applied directly to soil, where determining the viability of the eggs found is crucial. That is a mistake to have such a short incubation period (as indicated in the standards) as it may lead to laboratories giving erroneous results. Therefore, it is suggested to significantly extend the incubation period in order to increase the reliability of the obtained results.

At the same time, it seems that methods of assessing eggs viability based on a single assessment of the integrity of the internal structures of the eggs (without incubation) should be rejected for such purposes. Our study suggests that viability assessment carried out in this way may be flawed.

## Figures and Tables

**Table 1:** The average percentages of *A. suum* eggs of questionable viability (QE), containing live larvae (LE) and classified as dead (DE) isolated from the uteri of adult female roundworms observed in subsequent weeks of incubation.

Incubation Time [Week]	Eggs Category								
	QE			LE			DE		
	Avg (%) *	Range (±SD **)	*p*- Value ***	Avg (%) *	Range (±SD **)	*p*- Value ***	Avg (%) *	Range (±SD **)	*p*- Value ***
0	98	1.00 ± 0.00	<0.01	0	0–0 ± 0.00	<0.01	2	1–4 ± 1.00	<0.01
1	94	1.42 ± 0.00	<0.01	0	0–0 ± 0.00	<0.01	6	5–9 ± 1.42	<0.01
2	9	0.58 ± 0.00	<0.01	84	82–87 ± 1.37	<0.01	7	3–9 ± 1.68	<0.01
3	0	0 ± 0.00	<0.01	94	92–96 ± 1.31	<0.01	6	4–8 ± 1.31	<0.01
4	0	0 ± 0.00	<0.01	84	78–87 ± 2.94	<0.01	16	13–22 ± 2.94	<0.01
5	0	0 ± 0.00	<0.01	82	79–85 ± 2.15	<0.01	18	15–21 ± 2.15	<0.01
6	0	0 ± 0.00	<0.01	66	63–70 ± 2.31	<0.01	34	30–37 ± 2.31	<0.01
7	0	0 ± 0.00	<0.01	47	41–50 ± 2.71	<0.01	53	50–59 ± 2.71	<0.01
8	0	0 ± 0.00	<0.01	26	23–28 ± 1.38	<0.01	74	72–77 ± 1.38	<0.01
9	0	0 ± 0.00	<0.01	19	17–20 ± 0.98	<0.01	81	80–83 ± 0.98	<0.01
10	0	0 ± 0.00	<0.01	7	4–10 ± 1.83	<0.01	93	90–96 ± 1.83	<0.01
11	0	0 ± 0.00	<0.01	2	0–5 ± 1.42	<0.01	98	95–100 ± 1.42	<0.01
12	0	0 ± 0.00	-	0	0–1 ± 0.39	-	100	99–100 ± 0.39	-

* Avg—Average percentage of eggs; ** SD—standard deviation; *** *p*-value—statistical significance.

**Table 2:** The average percentages of *A. suum* eggs of questionable viability (QE), containing live larvae (LE) and classified as dead (DE) obtained from pig faeces, observed in subsequent weeks of incubation.

Incubation Time [Week]	Eggs Category								
	QE			LE			DE		
	Avg (%) *	Range (±SD **)	*p*- Value ***	Avg (%) *	Range (±SD **)	*p*- Value ***	Avg (%) *	Range (±SD **)	*p*- Value ***
0	90	87–97 ± 2.82	<0.01	0	0–0 ± 0.00	<0.01	10	3–13 ± 2.82	<0.01
1	76	72–80 ± 2.66	<0.01	0	0–0 ± 0.00	<0.01	24	20–28 ± 2.66	<0.01
2	70	66–73 ± 2.02	<0.01	0	0–0 ± 0.00	<0.01	30	27–34 ± 2.02	<0.01
3	21	14–25 ± 3.18	<0.01	42	39–49 ± 3.36	<0.01	37	35–40 ± 1.64	<0.01
4	18	14–24 ± 4.52	<0.01	46	40–51 ± 4.38	<0.01	36	35–38 ± 0.94	<0.01
5	14	10–18 ± 2.38	<0.01	52	49–55 ± 1.78	<0.01	34	31–39 ± 1.91	<0.01
6	10	8–13 ± 1.93	<0.01	40	37–43 ± 1.91	<0.01	50	47–53 ± 1.97	<0.01
7	8	6–10 ± 1.68	<0.01	29	25–34 ± 2.21	<0.01	63	59–66 ± 2.27	<0.01
8	0	0–0 ± 0.00	<0.01	15	12–20 ± 2.09	<0.01	85	80–88 ± 2.09	<0.01
9	0	0–2 ± 0.58	<0.01	8	6–11 ± 1.70	<0.01	92	89–94 ± 1.56	<0.01
10	0	0–0 ± 0.00	<0.01	5	3–7 ± 1.30	<0.01	95	93–97 ± 1.30	<0.01
11	0	0–0 ± 0.00	<0.01	2	1–3 ± 0.78	<0.01	98	97–99 ± 0.78	<0.01
12	0	0–0 ± 0.00	-	0	0–2 ± 0.65	-	100	98–100 ± 0.65	-

* Avg—Average percentage of eggs; ** SD—standard deviation; *** *p*-value—statistical significance.

**Table 3:** The average percentages of *A. suum* eggs of questionable viability (QE), containing live larvae (LE) and classified as dead (DE), obtained from sewage sludge, observed in subsequent weeks of incubation.

Incubation Time [Week]	Eggs Category								
	QE			LE			DE		
	Avg (%) *	Range (±SD **)	*p*- Value ***	Avg (%) *	Range (±SD **)	*p*- Value ***	Avg (%) *	Range (±SD **)	*p*- Value ***
0	79	66–92 ± 8.98	<0.01	0	0–0 ± 0.00	0.085	21	8–34 ± 8.98	<0.01
1	70	54–76 ± 8.21	<0.01	0	0–0 ± 0.00	0.085	30	24–46 ± 8.21	<0.01
2	59	35–68 ± 12.89	<0.01	0	0–0 ± 0.00	0.085	41	32–65 ± 12.89	<0.01
3	41	28–55 ± 8.68	<0.01	0	0–0 ± 0.00	0.085	59	45–72 ± 8.67	<0.01
4	22	13–37 ± 9.01	0.253	2	0–5 ± 1.88	0.638	76	58–87 ± 10.85	<0.01
5	14	4–21 ± 5.85	<0.01	3	0–10 ± 3.78	0.041	83	72–96 ± 8.58	<0.01
6	9	4–16 ± 4.12	<0.01	3	0–10 ± 3.78	0.041	88	78–96 ± 6.88	<0.01
7	6	2–11 ± 3.51	<0.01	3	0–10 ± 3.78	0.041	91	81–98 ± 6.53	<0.01
8	4	0–8 ± 2.71	<0.01	3	0–10 ± 3.78	0.041	93	86–100 ± 5.41	<0.01
9	3	0–7 ± 2.57	<0.01	2	0–10 ± 3.74	0.178	95	90–99 ± 3.41	<0.01
10	1	0–3 ± 1.16	<0.01	1	0–5 ± 1.91	0.917	98	95–100 ± 1.72	<0.01
11	1	0–4 ± 1.42	<0.01	0	0–0 ± 0.00	0.085	99	96–100 ± 1.42	<0.01
12	0	0–0 ± 0.00	-	0	0–2 ± 0.65	-	99	97–100 ± 1.03	-

* Avg—Average percentage of eggs; ** SD—standard deviation; *** *p*-value—statistical significance.

## Data Availability

The original contributions presented in this study are included in the article/Supplementary Material. Further inquiries can be directed to the corresponding author.

## Supplementary Materials

The following supporting information can be downloaded at: https://www.mdpi.com/article/10.3390/pathogens14101070/s1, Table S1: Statistical significance of differences in the percentage of *A. suum* eggs with normal structure (QE) obtained from sewage sludge (S)—Tukey post hoc test; Table S2: Statistical significance of differences in the percentage of *A. suum* eggs with normal structure (QE) obtained from pig faeces (F)—Tukey post hoc test; Table S3: Statistical significance of differences in the percentage of *A. suum* eggs with normal structure (QE) isolated from the uteri of adult female roundworms (U)—Tukey post hoc test; Table S4: Statistical significance of differences in the percentage of *A. suum* eggs containing live larvae (LE) obtained from sewage sludge (S)—Tukey post hoc test; Table S5: Statistical significance of differences in the percentage of *A. suum* eggs containing live larvae (LE) obtained from pig faeces (F)—Tukey post hoc test; Table S6: Statistical significance of differences in the percentage of *A. suum* eggs containing live larvae (LE) isolated from the uteri of adult female roundworms (U)—Tukey post hoc test; Table S7: Statistical significance of differences in the percentage of dead *A. suum* eggs (DE) obtained from sewage sludge (S)—Tukey post hoc test; Table S8: Statistical significance of differences in the percentage of dead *A. suum* eggs (DE) obtained from pig faeces (F)—Tukey post hoc test; Table S9: Statistical significance of differences in the percentage of dead *A. suum* eggs (DE) isolated from the uteri of adult female roundworms (U)—Tukey post hoc test; Supplementary Table S10: A summary of the statistical value (Z-scores and t-value) for *A. suum* eggs (QE, LE and DE) obtained from the uterus, isolated from faeces and sewage sludge, over 12 weeks of incubation.
