# Supplementary material for: Influence of Incubation Time for Viability Assessment of Ascaris suum Eggs—Attempts to Optimise the Parasitological Examination
Source: Pathogens. 2025 Oct 21;14(10):1070. doi: 10.3390/pathogens14101070 (PMC12567136; doi:10.3390/pathogens14101070)
Supplement: Supplementary file 1 [file pathogens-14-01070-s001.zip › pathogens-3843182-supplementary.pdf]

Supplementary Table S1. Statistical significance of differences in the percentage of *A. suum* eggs with normal structure (QE) obtained from sewage sludge (S) – Tukey post hoc test.

Weeks	Mean (%) QE eggs												
	79.250	70.000	58.833	40.833	21.750	14.583	9.8333	5.8333	3.5833	2.0000	1.0833	1.2500	1.1667
	p-value												
**0**		0.00005	0.00004	0.00004	0.00004	0.00004	0.00004	0.00004	0.00004	0.00004	0.00004	0.00004	0.00004
**I**	0.00005		0.00004	0.00004	0.00004	0.00004	0.00004	0.00004	0.00004	0.00004	0.00004	0.00004	0.00004
**II**	0.00004	0.00004		0.00004	0.00004	0.00004	0.00004	0.00004	0.00004	0.00004	0.00004	0.00004	0.00004
**III**	0.00004	0.00004	0.00004		0.00004	0.00004	0.00004	0.00004	0.00004	0.00004	0.00004	0.00004	0.00004
**IV**	0.00004	0.00004	0.00004	0.00004		0.00729	0.00004	0.00004	0.00004	0.00004	0.00004	0.00004	0.00004
**V**	0.00004	0.00004	0.00004	0.00004	0.00729		0.59454	0.00010	0.00004	0.00004	0.00004	0.00004	0.00004
**VI**	0.00004	0.00004	0.00004	0.00004	0.00004	0.59454		0.90904	0.06206	0.00117	0.00010	0.00014	0.00012
**VII**	0.00004	0.00004	0.00004	0.00004	0.00004	0.00010	0.90904		1.00000	0.94552	0.59454	0.68023	0.63789
**VIII**	0.00004	0.00004	0.00004	0.00004	0.00004	0.00004	0.06206	1.00000		1.00000	0.99998	1.00000	0.99999
**IX**	0.00004	0.00004	0.00004	0.00004	0.00004	0.00004	0.00117	0.94552	1.00000		1.00000	1.00000	1.00000
**X**	0.00004	0.00004	0.00004	0.00004	0.00004	0.00004	0.00010	0.59454	0.99998	1.00000		1.00000	1.00000
**XI**	0.00004	0.00004	0.00004	0.00004	0.00004	0.00004	0.00014	0.68023	1.00000	1.00000	1.00000		1.00000
**XII**	0.00004	0.00004	0.00004	0.00004	0.00004	0.00004	0.00012	0.63789	0.99999	1.00000	1.00000	1.00000	

Read colour – statistical significant

Black colour – no statistical significant

Supplementary Table S2. Statistical significance of differences in the percentage of *A. suum* eggs with normal structure (QE) obtained from pig faeces (F) – Tukey post hoc test.

Weeks	Mean (%) QE eggs												
	89.833	76.000	69.333	20.500	18.583	14.250	10.583	7.9167	0.0000	0.0000	0.0000	0.0000	0.0000
	p-value												
**0**		0.00004	0.00004	0.00004	0.00004	0.00004	0.00004	0.00004	0.00004	0.00004	0.00004	0.00004	0.00004
**I**	0.00004		0.02489	0.00004	0.00004	0.00004	0.00004	0.00004	0.00004	0.00004	0.00004	0.00004	0.00004
**II**	0.00004	0.02489		0.00004	0.00004	0.00004	0.00004	0.00004	0.00004	0.00004	0.00004	0.00004	0.00004
**III**	0.00004	0.00004	0.00004		1.00000	0.06206	0.00004	0.00004	0.00004	0.00004	0.00004	0.00004	0.00004
**IV**	0.00004	0.00004	0.00004	1.00000		0.79583	0.00072	0.00004	0.00004	0.00004	0.00004	0.00004	0.00004
**V**	0.00004	0.00004	0.00004	0.06206	0.79583		0.97001	0.05214	0.00004	0.00004	0.00004	0.00004	0.00004
**VI**	0.00004	0.00004	0.00004	0.00004	0.00072	0.97001		0.99993	0.00004	0.00004	0.00004	0.00004	0.00004
**VII**	0.00004	0.00004	0.00004	0.00004	0.00004	0.05214	0.99993		0.00092	0.00148	0.00092	0.00092	0.00092
**VIII**	0.00004	0.00004	0.00004	0.00004	0.00004	0.00004	0.00004	0.00092		1.00000	1.00000	1.00000	1.00000
**IX**	0.00004	0.00004	0.00004	0.00004	0.00004	0.00004	0.00004	0.00148	1.00000		1.00000	1.00000	1.00000
**X**	0.00004	0.00004	0.00004	0.00004	0.00004	0.00004	0.00004	0.00092	1.00000	1.00000		1.00000	1.00000
**XI**	0.00004	0.00004	0.00004	0.00004	0.00004	0.00004	0.00004	0.00092	1.00000	1.00000	1.00000		1.00000
**XII**	0.00004	0.00004	0.00004	0.00004	0.00004	0.00004	0.00004	0.00092	1.00000	1.00000	1.00000	1.00000	

Read colour – statistical significant

Black colour – no statistical significant

Supplementary Table S3. Statistical significance of differences in the percentage of *A. suum* eggs with normal structure (QE) isolated from the uteri of adult female roundworms (U) – Tukey post hoc test.

Weeks	Mean (%) QE eggs												
	97.917	93.750	9.1667	0.0000	0.0000	0.0000	0.0000	0.0000	0.0000	0.0000	0.0000	0.0000	0.0000
	p-value												
**0**		0.85911	0.00004	0.00004	0.00004	0.00004	0.00004	0.00004	0.00004	0.00004	0.00004	0.00004	0.00004
**I**	0.85911		0.00004	0.00004	0.00004	0.00004	0.00004	0.00004	0.00004	0.00004	0.00004	0.00004	0.00004
**II**	0.00004	0.00004		0.00005	0.00005	0.00005	0.00005	0.00005	0.00005	0.00005	0.00005	0.00005	0.00005
**III**	0.00004	0.00004	0.00005		1.00000	1.00000	1.00000	1.00000	1.00000	1.00000	1.00000	1.00000	1.00000
**IV**	0.00004	0.00004	0.00005	1.00000		1.00000	1.00000	1.00000	1.00000	1.00000	1.00000	1.00000	1.00000
**V**	0.00004	0.00004	0.00005	1.00000	1.00000		1.00000	1.00000	1.00000	1.00000	1.00000	1.00000	1.00000
**VI**	0.00004	0.00004	0.00005	1.00000	1.00000	1.00000		1.00000	1.00000	1.00000	1.00000	1.00000	1.00000
**VII**	0.00004	0.00004	0.00005	1.00000	1.00000	1.00000	1.00000		1.00000	1.00000	1.00000	1.00000	1.00000
**VIII**	0.00004	0.00004	0.00005	1.00000	1.00000	1.00000	1.00000	1.00000		1.00000	1.00000	1.00000	1.00000
**IX**	0.00004	0.00004	0.00005	1.00000	1.00000	1.00000	1.00000	1.00000	1.00000		1.00000	1.00000	1.00000
**X**	0.00004	0.00004	0.00005	1.00000	1.00000	1.00000	1.00000	1.00000	1.00000	1.00000		1.00000	1.00000
**XI**	0.00004	0.00004	0.00005	1.00000	1.00000	1.00000	1.00000	1.00000	1.00000	1.00000	1.00000		1.00000
**XII**	0.00004	0.00004	0.00005	1.00000	1.00000	1.00000	1.00000	1.00000	1.00000	1.00000	1.00000	1.00000	

Read colour – statistical significant

Black colour – no statistical significant

Supplementary Table S4. Statistical significance of differences in the percentage of *A. suum* eggs containing live larvae (LE) obtained from sewage sludge (S) – Tukey post hoc test.

	Mean (%) LE eggs												
**Weeks**	0.0000	0.0000	0.0000	0.0000	1.5000	2.5833	2.5833	2.5833	2.5833	2.2500	1.2500	0.0000	0.0000
	p-value												
**0**		1.00000	1.00000	1.00000	0.99953	0.46825	0.46825	0.46825	0.46825	0.78991	0.99999	1.00000	1.00000
**I**	1.00000		1.00000	1.00000	0.99953	0.46825	0.46825	0.46825	0.46825	0.78991	0.99999	1.00000	1.00000
**II**	1.00000	1.00000		1.00000	0.99953	0.46825	0.46825	0.46825	0.46825	0.78991	0.99999	1.00000	1.00000
**III**	1.00000	1.00000	1.00000		0.99953	0.46825	0.46825	0.46825	0.46825	0.78991	0.99999	1.00000	1.00000
**IV**	0.99953	0.99953	0.99953	0.99953		1.00000	1.00000	1.00000	1.00000	1.00000	1.00000	0.99953	0.99953
**V**	0.46825	0.46825	0.46825	0.46825	1.00000		1.00000	1.00000	1.00000	1.00000	0.99997	0.46825	0.46825
**VI**	0.46825	0.46825	0.46825	0.46825	1.00000	1.00000		1.00000	1.00000	1.00000	0.99997	0.46825	0.46825
**VII**	0.46825	0.46825	0.46825	0.46825	1.00000	1.00000	1.00000		1.00000	1.00000	0.99997	0.46825	0.46825
**VIII**	0.46825	0.46825	0.46825	0.46825	1.00000	1.00000	1.00000	1.00000		1.00000	0.99997	0.46825	0.46825
**IX**	0.78991	0.78991	0.78991	0.78991	1.00000	1.00000	1.00000	1.00000	1.00000		1.00000	0.78991	0.78991
**X**	0.99999	0.99999	0.99999	0.99999	1.00000	0.99997	0.99997	0.99997	0.99997	1.00000		0.99999	0.99999
**XI**	1.00000	1.00000	1.00000	1.00000	0.99953	0.46825	0.46825	0.46825	0.46825	0.78991	0.99999		1.00000
**XII**	1.00000	1.00000	1.00000	1.00000	0.99953	0.46825	0.46825	0.46825	0.46825	0.78991	0.99999	1.00000	

Black colour – no statistical significant

Supplementary Table S5. Statistical significance of differences in the percentage of *A. suum* eggs containing live larvae (LE) obtained from pig faeces (F) – Tukey post hoc test.

	Mean (%) LE eggs												
**Weeks**	0.0000	0.0000	0.0000	42.250	45.583	51.500	39.750	29.167	15.250	8.1667	4.6667	1.6667	0.3333
	p-value												
**0**		1.00000	1.00000	0.00004	0.00004	0.00004	0.00004	0.00004	0.00004	0.00004	0.00006	0.99631	1.00000
**I**	1.00000		1.00000	0.00004	0.00004	0.00004	0.00004	0.00004	0.00004	0.00004	0.00006	0.99631	1.00000
**II**	1.00000	1.00000		0.00004	0.00004	0.00004	0.00004	0.00004	0.00004	0.00004	0.00006	0.99631	1.00000
**III**	0.00004	0.00004	0.00004		0.04139	0.00004	0.55223	0.00004	0.00004	0.00004	0.00004	0.00004	0.00004
**IV**	0.00004	0.00004	0.00004	0.04139		0.00004	0.00004	0.00004	0.00004	0.00004	0.00004	0.00004	0.00004
**V**	0.00004	0.00004	0.00004	0.00004	0.00004		0.00004	0.00004	0.00004	0.00004	0.00004	0.00004	0.00004
**VI**	0.00004	0.00004	0.00004	0.55223	0.00004	0.00004		0.00004	0.00004	0.00004	0.00004	0.00004	0.00004
**VII**	0.00004	0.00004	0.00004	0.00004	0.00004	0.00004	0.00004		0.00004	0.00004	0.00004	0.00004	0.00004
**VIII**	0.00004	0.00004	0.00004	0.00004	0.00004	0.00004	0.00004	0.00004		0.00004	0.00004	0.00004	0.00004
**IX**	0.00004	0.00004	0.00004	0.00004	0.00004	0.00004	0.00004	0.00004	0.00004		0.01987	0.00004	0.00004
**X**	0.00006	0.00006	0.00006	0.00004	0.00004	0.00004	0.00004	0.00004	0.00004	0.01987		0.14762	0.00025
**XI**	0.99631	0.99631	0.99631	0.00004	0.00004	0.00004	0.00004	0.00004	0.00004	0.00004	0.14762		0.99997
**XII**	1.00000	1.00000	1.00000	0.00004	0.00004	0.00004	0.00004	0.00004	0.00004	0.00004	0.00025	0.99997	

Read colour – statistical significant

Black colour – no statistical significant

Supplementary Table S6. Statistical significance of differences in the percentage of *A. suum* eggs containing live larvae (LE) isolated from the uteri of adult female roundworms (U) – Tukey post hoc test.

	Mean (%) LE eggs												
**Weeks**	0.0000	0.0000	84.333	94.417	83.583	81.500	65.667	47.083	25.583	18.667	7.0833	1.7500	0.1666
	p-value												
**0**		1.00000	0.00004	0.00004	0.00004	0.00004	0.00004	0.00004	0.00004	0.00004	0.00004	0.99139	1.00000
**I**	1.00000		0.00004	0.00004	0.00004	0.00004	0.00004	0.00004	0.00004	0.00004	0.00004	0.99139	1.00000
**II**	0.00004	0.00004		0.00004	1.00000	0.24950	0.00004	0.00004	0.00004	0.00004	0.00004	0.00004	0.00004
**III**	0.00004	0.00004	0.00004		0.00004	0.00004	0.00004	0.00004	0.00004	0.00004	0.00004	0.00004	0.00004
**IV**	0.00004	0.00004	1.00000	0.00004		0.90232	0.00004	0.00004	0.00004	0.00004	0.00004	0.00004	0.00004
**V**	0.00004	0.00004	0.24950	0.00004	0.90232		0.00004	0.00004	0.00004	0.00004	0.00004	0.00004	0.00004
**VI**	0.00004	0.00004	0.00004	0.00004	0.00004	0.00004		0.00004	0.00004	0.00004	0.00004	0.00004	0.00004
**VII**	0.00004	0.00004	0.00004	0.00004	0.00004	0.00004	0.00004		0.00004	0.00004	0.00004	0.00004	0.00004
**VIII**	0.00004	0.00004	0.00004	0.00004	0.00004	0.00004	0.00004	0.00004		0.00004	0.00004	0.00004	0.00004
**IX**	0.00004	0.00004	0.00004	0.00004	0.00004	0.00004	0.00004	0.00004	0.00004		0.00004	0.00004	0.00004
**X**	0.00004	0.00004	0.00004	0.00004	0.00004	0.00004	0.00004	0.00004	0.00004	0.00004		0.00004	0.00004
**XI**	0.99139	0.99139	0.00004	0.00004	0.00004	0.00004	0.00004	0.00004	0.00004	0.00004	0.00004		0.99860
**XII**	1.00000	1.00000	0.00004	0.00004	0.00004	0.00004	0.00004	0.00004	0.00004	0.00004	0.00004	0.99860	

Read colour – statistical significant

Black colour – no statistical significant

Supplementary Table S7. Statistical significance of differences in the percentage of dead *A. suum* eggs (DE) obtained from sewage sludge (S) – Tukey post hoc test.

**Weeks**	**Mean (%) DE eggs**												
	20.750	30.000	41.167	59.250	76.750	83.667	87.333	91.583	93.833	95.167	97.667	98.750	98.833
	**p-value**												
**0**		0.00046	0.00004	0.00004	0.00004	0.00004	0.00004	0.00004	0.00004	0.00004	0.00004	0.00004	0.00004
**I**	0.00046		0.00004	0.00004	0.00004	0.00004	0.00004	0.00004	0.00004	0.00004	0.00004	0.00004	0.00004
**II**	0.00004	0.00004		0.00004	0.00004	0.00004	0.00004	0.00004	0.00004	0.00004	0.00004	0.00004	0.00004
**III**	0.00004	0.00004	0.00004		0.00004	0.00004	0.00004	0.00004	0.00004	0.00004	0.00004	0.00004	0.00004
**IV**	0.00004	0.00004	0.00004	0.00004		0.08429	0.00005	0.00004	0.00004	0.00004	0.00004	0.00004	0.00004
**V**	0.00004	0.00004	0.00004	0.00004	0.08429		0.99605	0.01172	0.00007	0.00004	0.00004	0.00004	0.00004
**VI**	0.00004	0.00004	0.00004	0.00004	0.00005	0.99605		0.95957	0.16543	0.01407	0.00005	0.00004	0.00004
**VII**	0.00004	0.00004	0.00004	0.00004	0.00004	0.01172	0.95957		1.00000	0.99741	0.29348	0.05382	0.04602
**VIII**	0.00004	0.00004	0.00004	0.00004	0.00004	0.00007	0.16543	1.00000		1.00000	0.99146	0.79526	0.76349
**IX**	0.00004	0.00004	0.00004	0.00004	0.00004	0.00004	0.01407	0.99741	1.00000		1.00000	0.99741	0.99605
**X**	0.00004	0.00004	0.00004	0.00004	0.00004	0.00004	0.00005	0.29348	0.99146	1.00000		1.00000	1.00000
**XI**	0.00004	0.00004	0.00004	0.00004	0.00004	0.00004	0.00004	0.05382	0.79526	0.99741	1.00000		1.00000
**XII**	0.00004	0.00004	0.00004	0.00004	0.00004	0.00004	0.00004	0.04602	0.76349	0.99605	1.00000	1.00000	

Read colour – statistical significant

Black colour – no statistical significant

Supplementary Table S8. Statistical significance of differences in the percentage of dead *A. suum* eggs (DE) obtained from pig faeces (F) – Tukey post hoc test.

**Weeks**	**Mean (%) DE eggs**												
	10.167	24.000	30.667	37.250	35.833	34.250	49.667	62.917	84.750	91.667	95.333	98.333	99.667
	**p-value**												
**0**		0.00004	0.00004	0.00004	0.00004	0.00004	0.00004	0.00004	0.00004	0.00004	0.00004	0.00004	0.00004
**I**	0.00004		0.12779	0.00004	0.00004	0.00006	0.00004	0.00004	0.00004	0.00004	0.00004	0.00004	0.00004
**II**	0.00004	0.12779		0.14568	0.69424	0.99741	0.00004	0.00004	0.00004	0.00004	0.00004	0.00004	0.00004
**III**	0.00004	0.00004	0.14568		1.00000	0.99994	0.00004	0.00004	0.00004	0.00004	0.00004	0.00004	0.00004
**IV**	0.00004	0.00004	0.69424	1.00000		1.00000	0.00004	0.00004	0.00004	0.00004	0.00004	0.00004	0.00004
**V**	0.00004	0.00006	0.99741	0.99994	1.00000		0.00004	0.00004	0.00004	0.00004	0.00004	0.00004	0.00004
**VI**	0.00004	0.00004	0.00004	0.00004	0.00004	0.00004		0.00004	0.00004	0.00004	0.00004	0.00004	0.00004
**VII**	0.00004	0.00004	0.00004	0.00004	0.00004	0.00004	0.00004		0.00004	0.00004	0.00004	0.00004	0.00004
**VIII**	0.00004	0.00004	0.00004	0.00004	0.00004	0.00004	0.00004	0.00004		0.08429	0.00005	0.00004	0.00004
**IX**	0.00004	0.00004	0.00004	0.00004	0.00004	0.00004	0.00004	0.00004	0.08429		0.99605	0.12779	0.00975
**X**	0.00004	0.00004	0.00004	0.00004	0.00004	0.00004	0.00004	0.00004	0.00005	0.99605		0.99994	0.94782
**XI**	0.00004	0.00004	0.00004	0.00004	0.00004	0.00004	0.00004	0.00004	0.00004	0.12779	0.99994		1.00000
**XII**	0.00004	0.00004	0.00004	0.00004	0.00004	0.00004	0.00004	0.00004	0.00004	0.00975	0.94782	1.00000	

Read colour – statistical significant

Black colour – no statistical significant

Supplementary Table S9. Statistical significance of differences in the percentage of dead *A. suum* eggs (DE) isolated from the uteri of adult female roundworms (U) – Tukey post hoc test.

	**Mean (%) DE eggs**												
**Weeks**	2.0833	6.2500	6.5000	5.5833	16.417	18.500	34.333	52.917	74.417	81.333	92.917	98.250	99.833
	**p-value**												
**0**		0.96922	0.93376	0.99834	0.00004	0.00004	0.00004	0.00004	0.00004	0.00004	0.00004	0.00004	0.00004
**I**	0.96922		1.00000	1.00000	0.00007	0.00004	0.00004	0.00004	0.00004	0.00004	0.00004	0.00004	0.00004
**II**	0.93376	1.00000		1.00000	0.00010	0.00004	0.00004	0.00004	0.00004	0.00004	0.00004	0.00004	0.00004
**III**	0.99834	1.00000	1.00000		0.00004	0.00004	0.00004	0.00004	0.00004	0.00004	0.00004	0.00004	0.00004
**IV**	0.00004	0.00007	0.00010	0.00004		1.00000	0.00004	0.00004	0.00004	0.00004	0.00004	0.00004	0.00004
**V**	0.00004	0.00004	0.00004	0.00004	1.00000		0.00004	0.00004	0.00004	0.00004	0.00004	0.00004	0.00004
**VI**	0.00004	0.00004	0.00004	0.00004	0.00004	0.00004		0.00004	0.00004	0.00004	0.00004	0.00004	0.00004
**VII**	0.00004	0.00004	0.00004	0.00004	0.00004	0.00004	0.00004		0.00004	0.00004	0.00004	0.00004	0.00004
**VIII**	0.00004	0.00004	0.00004	0.00004	0.00004	0.00004	0.00004	0.00004		0.08429	0.00004	0.00004	0.00004
**IX**	0.00004	0.00004	0.00004	0.00004	0.00004	0.00004	0.00004	0.00004	0.08429		0.00004	0.00004	0.00004
**X**	0.00004	0.00004	0.00004	0.00004	0.00004	0.00004	0.00004	0.00004	0.00004	0.00004		0.61958	0.08429
**XI**	0.00004	0.00004	0.00004	0.00004	0.00004	0.00004	0.00004	0.00004	0.00004	0.00004	0.61958		1.00000
**XII**	0.00004	0.00004	0.00004	0.00004	0.00004	0.00004	0.00004	0.00004	0.00004	0.00004	0.08429	1.00000	

Read colour – statistical significant

Black colour – no statistical significant

Supplementary Table S10. A summary of the statistical value (Z-scores and t-value) for *A. suum* eggs (QE, LE and DE) obtained from the uterus, isolated from faeces and sewage sludge, over 12 weeks of incubation.

Incubation time [week]	Eggs category																	
	Uterus						Pig faeces						Sewage sludge					
	QE		LE		DE		QE		LE		DE		QE		LE		DE	
	Z-scores	t-value	Z-scores	t-value	Z-scores	t-value	Z-scores	t-value	Z-scores	t-value	Z-scores	t-value	Z-scores	t-value	Z-scores	t-value	Z-scores	t-value
**0**	82.47	585.9	-39.22	-82.76	-43.25	-86.85	66.21	109.47	-18.33	-32.99	-47.87	-92.59	55.40	30.34	-1.179	-1.733	-54.23	-26.24
**1**	78.30	556.3	-39.22	-82.76	-39.08	-78.48	52.37	86.59	-18.33	-32.99	-34.04	-65.83	46.15	25.27	-1.179	-1.733	-44.98	-21.76
**2**	-6.28	-44.6	45.12	95.20	-38.83	-77.98	45.71	75.57	-18.33	-32.99	-27.37	-52.94	34.99	19.16	-1.179	-1.733	-33.81	-16.36
**3**	-15.45	-109.8	55.20	116.48	-39.75	-79.82	-3.13	-5.17	23.92	43.04	-20.79	-40.21	16.99	9.30	-1.179	-1.733	-15.73	-7.61
**4**	-15.45	-109.8	44.37	93.62	-28.92	-58.07	-5.04	-8.34	27.25	49.04	-22.21	-42.95	-2.10	-1.15	0.321	0.471	1.77	0.86
**5**	-15.45	-109.8	42.28	89.22	-26.83	-53.88	-9.38	-15.51	33.17	59.69	-23.79	-46.01	-9.26	-5.07	1.404	2.063	8.69	4.20
**6**	-15.45	-109.8	26.45	55.81	-11.00	-22.09	-13.04	-21.57	21.42	38.54	-8.37	-16.19	-14.01	-7.67	1.404	2.063	12.35	5.98
**7**	-15.45	-109.8	7.87	16.60	7.58	15.23	-15.71	-25.98	10.83	19.50	4.88	9.43	-18.01	-9.86	1.404	2.063	16.60	8.03
**8**	-15.45	-109.8	-13.63	-28.77	29.08	58.40	-23.63	-39.07	-3.08	-5.55	26.71	51.66	-20.26	-11.10	1.404	2.063	18.85	9.12
**9**	-15.45	-109.8	-20.55	-43.37	36.00	72.29	-23.46	-38.79	-10.17	-18.30	33.63	65.04	-21.85	-11.96	1.071	1.573	20.19	9.77
**10**	-15.45	-109.8	-32.13	-67.81	47.58	95.55	-23.63	-39.07	-13.67	-24.59	37.29	72.13	-22.76	-12.46	0.071	0.104	22.69	10.98
**11**	-15.45	-109.8	-37.47	-79.06	52.92	106.26	-23.63	-39.07	-16.67	-29.99	40.29	77.93	-22.60	-12.37	-1.179	-1.733	23.77	11.50
**12**	-	-	-	-	-	-	-	-	-	-	-	-	-	-	-	-	-	-

* Avg - Average percentage of eggs; ** SD - standard deviation; *** p-value – statistical significance
